# Estimating the long-term impact of a prophylactic human papillomavirus 16/18 vaccine on the burden of cervical cancer in the UK

**DOI:** 10.1038/sj.bjc.6603501

**Published:** 2006-12-05

**Authors:** M Kohli, N Ferko, A Martin, E L Franco, D Jenkins, S Gallivan, C Sherlaw-Johnson, M Drummond

**Affiliations:** 1 Health Economics and Outcomes Research, i3 Innovus, Burlington, Ontario, Canada; 2 Health Outcomes, GlaxoSmithKline, Uxbridge, Middlesex, UK; 3 Departments of Epidemiology and Oncology, McGill University, Montreal, Quebec, Canada; 4 Clinical Development, GlaxoSmithKline Biologicals, Rixensart, Belgium; 5 Clinical Operational Research Unit, University College London, London, UK; 6 i3 Innovus, Uxbridge, Middlesex, UK; 7 Centre for Health Economics, University of York, Heslington, York, UK

**Keywords:** cervical cancer, human papillomavirus, vaccination, mathematical model, screening

## Abstract

To predict the public health impact on cervical disease by introducing human papillomavirus (HPV) vaccination in the United Kingdom, we developed a mathematical model that can be used to reflect the impact of vaccination in different countries with existing screening programmes. Its use is discussed in the context of the United Kingdom. The model was calibrated with published data. The impact of vaccination on cervical cancer and deaths, precancerous lesions and screening outcomes were estimated for a vaccinated cohort of 12-year-old girls, among which it is estimated that there would be a reduction of 66% in the prevalence of high-grade precancerous lesions and a 76% reduction in cervical cancer deaths. Estimates for various other measures of the population effects of vaccination are also presented. We concluded that it is feasible to forecast the potential effects of HPV vaccination in the context of an existing national screening programme. Results suggest a sizable reduction in the incidence of cervical cancer and related deaths. Areas for future research include investigation of the beneficial effects of HPV vaccination on infection transmission and epidemic dynamics, as well as HPV-related neoplasms in other sites.

Invasive cervical cancer (ICC) is the second most common cancer in women worldwide, with approximately 470 000 cases and 230 000 deaths annually (Ferlay *et al*, 2002). The causal role of human papillomavirus (HPV) in ICC is well established (Franco *et al*, 1999a), with at least 13 types associated with ICC (Cogliano *et al*, 2005). In the United Kingdom (UK), the cervical screening programme has substantially reduced ICC deaths in women born since 1950 (Peto *et al*, 2004a). However, ICC remains high for a developed country, and cervical intraepithelial neoplasia (CIN) 3 and HPV incidence have increased in the last decade (Kitchener *et al*, 2004; Peto *et al*, 2004b).

Globally, infection with HPV 16 and 18 accounts for more than 70% of ICC cases (Muñoz *et al.*, 2004). Two vaccines have been in development and both show high levels of efficacy against these types (Koutsky *et al*, 2002; Harper *et al*, 2004, 2006; Villa *et al*, 2005). A trial of one of these, a bi-valent HPV-16/18 vaccine, reported efficacy of 95.1% (95% CI: 63.5–99.3%) against persistent HPV-16/18 infection (Harper *et al*, 2004), and efficacy was maintained over 4.5 years (Harper *et al*, 2006). Protection against HPV types 31 (54.5% (95% CI: 11.5–77.7%)) and 45 (94.2% (95% CI: 63.3–99.9%)) was also shown. Before vaccine implementation, it is desirable to understand the long-term benefit using mathematical modelling.

There is a long history of modelling in cervical cancer (Sherlaw-Johnson *et al*, 1994, 1999; Jenkins *et al*, 1996; Karnon *et al*, 2004; Sherlaw-Johnson and Philips, 2004), and studies in the US (Kulasingham and Myers, 2003; Goldie *et al*, 2004; Taira *et al*, 2004) have found that HPV vaccination with screening is cost-effective. There have been no published analyses of HPV vaccination in the UK, where cervical disease and screening differs from the US. We developed a generic model that could be used to evaluate vaccine impact in different countries. In this paper, we report the use of this model in the UK.

## MATERIALS AND METHODS

### Model overview

**Figure 1:**
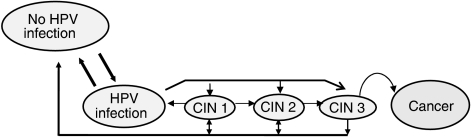
Simplified structure of the human papillomavirus (HPV) and cervical cancer natural history model. Model simulates the natural history of HPV infection and cervical carcinogenesis while incorporating the underlying type-specific HPV distribution within each stage of cervical disease, by use of a sequence of 6-month transitions among mutually exclusive health states. The probabilities governing each of these transitions are conditional on the type of HPV infection and age. Transitions to death owing to natural causes can occur from any health state in the model.

A Markov process model has been developed reflecting the natural history of type-specific HPV infection and progression of cervical lesions. The model is based on a set of mutually exclusive health states corresponding to HPV infection, CIN lesions and ICC (Figure 1). The aim of such modelling is to estimate the age-related probabilities that an individual would be in one of the states. The overall population effects of particular interventions are inferred by extrapolating these health-state probabilities over an entire birth cohort.

In computational terms, the model is composed of three modules. The natural history module reflects the natural history of disease without intervention. In parallel, the screening module incorporates screening practice by adjusting the health state probabilities corresponding to the proportion of women detected and treated for disease. The vaccination module is structured to allow the evaluation of the vaccination programme, in addition to screening practice, by altering transition rates that reflect the natural history of infection acquisition.

### Natural history

The natural history of cervical disease is modelled as a sequence of transitions, between mutually exclusive health states, occurring every 6 months. The transitions occur with probabilities that are age- and HPV type-specific. There are seven HPV categories in the model (oncogenic HPV types (16, 18, 31, 45, 52, other oncogenic) and low-risk HPV), where each category is subdivided into health states corresponding to ‘normal’ (un-infected and disease-free), four cervical disease states (HPV with no lesion, CIN 1, CIN 2 and CIN 3) and four ICC states (Federation of Gynaecology and Obstetrics (FIGO) Stage 1, Stage 2, Stage 3, Stage 4). The four FIGO stages are subdivided according to whether cancer is diagnosed (i.e., detected *vs* undetected cancer). Finally, there are additional health states corresponding to cervical cancer and noncervical cancer death.

It is assumed that in the absence of treatment, individuals can progress or regress from states up to CIN 3 and thereafter may progress to cervical cancer. Once in the cancer state, there is assumed to be potential progression through successive cancer states with no regression. Transition from the undetected to the detected cancer state is defined by the stage-dependent probability of developing symptoms. Further, these transitions are assumed to depend on whether a woman takes part in screening, reflecting the fact that cancer would be more likely to be detected with cytology. If detected, it is assumed that all cancers are treated and individuals remain in the same cancer state for their lifetime. Movement to either cervical cancer or noncervical cancer death is governed by cancer stage-related survival distribution, and competing all-cause mortality.

In addition to being HPV type-specific, the risk of acquiring infection and disease progression is age-specific, to account for age-related differences in behaviour and biological susceptibility. The potential role of natural immunity or age-related changes in the cervix has not been explicitly modelled; however, the model calibration process discussed below implicitly accounts for these factors.

### Screening

The screening module is based on a decision tree representing options available in the UK after an abnormal cytology test, including repeat cytology, diagnostic testing, more frequent cytological follow-up and treatment of diagnosed lesions.

The ages at which women are screened, coverage rates and observed screening practices are based on data reported from the Cervical Screening Programme (Department of Health Bulletin, 2004). Where data were not available, recommendations from UK screening guidelines were modelled (NHS, 2004). It was assumed that women were compliant with screening, where either testing or treatment was completed after abnormal diagnoses. After colposcopy and biopsy, women are assumed to either revert to regular screening again in 3 years, or increased screening within 1 year depending on the diagnosis. Treatment of CIN lesions was modelled using data from the National Health Service's cervical cancer screening guidelines (NHS, 2004) and the International Agency for Research on Cancer (IARC) (2005) where it is reported that the success rate of treating lesions is 90%. The sensitivity and specificity of cytology was obtained from a UK study (Cuzick *et al*, 1995). Colposcopy and biopsy test characteristics were obtained from a review of studies (Hopman *et al*, 1998; Mitchell *et al*, 1998). Details of screening inputs are provided in Table 1.

### Vaccination module

Vaccine efficacy is modelled by decreasing the probability of acquiring HPV infection in the different HPV strata. Vaccine efficacy could be varied over time, following vaccination, to incorporate waning of efficacy. The model can accommodate any pattern of vaccine waning and inclusion of booster shots. With a booster shot, it is assumed that vaccine efficacy is restored to original levels.

### Calibration

Calibration is the process of deriving estimates for quantities used within the model as the basis for its predictions. Some of these, such as screening coverage or stage-specific cancer survival, can be taken from the literature. Although there may be some uncertainty concerning such estimates, they at least provide a credible starting point for analysis. Other quantities used within the model, particularly some of the disease progression probabilities, are more difficult to estimate as there is a dearth of published literature on this topic. To estimate these, a process of calibration was carried out, by allowing transition probabilities to vary within established ranges, so that modelled predictions of key end points for unvaccinated girls matched currently available epidemiological data in the presence of screening. A comprehensive review of the literature was completed to determine plausible transition probability ranges for calibration (sample references in Table 2).

The following end points were used for calibration:

1. Age-specific HPV prevalence: data obtained from a population-based study of 6 462 girls in Manchester, between 1987 and 1993 (Peto *et al*, 2004b).
2. HPV type distribution: Mid-point of values (UK studies) of HPV-type distribution in normal cytology, low- and high-grade lesions and ICC for each HPV stratum (16, 18, 31, 45, 52, other oncogenic, low-risk) (Clifford *et al*, 2003a, 2003b, 2005a, 2005b).
3. CIN prevalence: age-specific prevalence patterns of CIN 1 or less (i.e., cytologically confirmed), CIN 2 and CIN 3 lesions (i.e., histologically confirmed) reported in Manchester study (Peto *et al*, 2004b). Overall CIN prevalence by lesion type reported in Department of Health Bulletin (2004).
4. Cancer incidence and mortality: age-specific rates (/100 000) from Office of National Statistics (2002a, 2002b). Historical incidence rates from IARC (Parkin, 1966).

Goodness-of-fit for HPV prevalence, cervical cancer incidence and mortality were measured using an average percentage deviation calculation (i.e., (observed – model predicted)/observed), weighted by: (1) age-specific disease rates and (2) age-specific number of cases expected. For CIN prevalence, goodness-of-fit was calculated using overall rates.

### Base-case analysis

We conducted an analysis to estimate the public health benefits associated with HPV-16/18 vaccination in the setting of cervical cancer screening in the UK, based on the conservative assumption that current screening practice does not change. For vaccinated and unvaccinated females, clinical events (i.e., HPV infections, CIN lesions, cervical cancer cases and deaths) were derived from the model for one birth cohort of girls from age of vaccination until a maximum of 110 years. Rates of screening events (abnormal cytology tests, biopsies, colposcopies, CIN treatments) were also estimated. A vaccination coverage rate of 100% was assumed.

To examine the protection afforded by vaccination, a cohort of 376 385 girls vaccinated at age 12 was modelled, representing one annual age cohort in the UK (ONS, 2003). The following assumptions were made based on published studies (Harper *et al*, 2004, 2006): (1) vaccination would reduce the probability of acquiring HPV 16/18 by 95%; (2) vaccination offers protection against other oncogenic types, reducing the probability of HPV 31 infection by 50%, and HPV 45 infection by 90%; (3) adolescents would receive three doses of the vaccine and be fully immunized after 1 year; and (4) vaccine efficacy does not wane over time. Results from the extended phase of a clinical trial (Harper *et al*, 2006) indicate that waning is not expected in the short term (e.g., up to 10 years). In the longer term, such waning could in principle, be countered by a booster.

### Sensitivity analysis

Alternative assumptions about vaccine efficacy and waning, vaccination coverage rate, vaccination age and HPV type distribution were explored using sensitivity analyses. Vaccine efficacy for HPV types 16 and 18 was varied from 90 to 100%. As cross-protection against types 31 and 45 has as yet only been demonstrated for incident infection, alternative scenarios where cross-protection was absent or waned were analysed. Waning of immunity against types 31 and 45 was assumed to occur in a linear manner to 0% of initial efficacy after 10 years, and was analysed with and without a booster at 10 years after initial vaccination. In the 4.5 years of follow-up in clinical trials, waning of efficacy against HPV types 16 and 18 have not been observed; therefore, it was assumed that waning would either not occur or would be addressed by boosters. The impact of vaccinating older and younger girls was also analysed (10 and 18 years). Also, as 100% vaccination coverage is likely not achievable in practice in the near term, an alternative vaccination coverage rate of 80% was explored, based on the coverage actually achieved in recent vaccination campaigns in adolescents (Wallace *et al*, 2004). Finally, given the variability of literature that exists on HPV type distribution in the UK, we also conducted an analysis that assumes a lower HPV 16/18 type distribution (i.e., 72%) in ICC, reported for Europe (*vs* 77% assumed for the UK).

## RESULTS

### Model calibration

**Figure 2:**
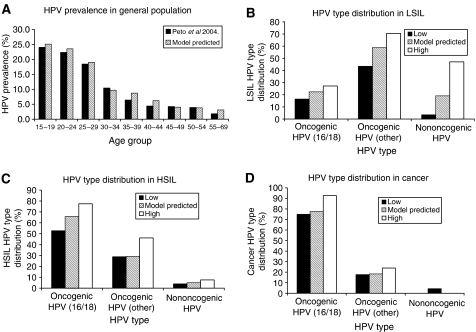
Comparison of model-predicted and observed data for HPV prevalence in the UK. (**A**) Age-specific HPV prevalence in the general population. (**B**) HPV type distribution within low-grade squamous intraepithelial lesions (LSIL). (**C**) HPV-type distribution within high-grade squamous intraepithelial lesions (HSIL)). (**D**) HPV-type distribution within cervical cancer. Oncogenic HPV types include all other oncogenic types except HPV types 16 and 18.

**Figure 3:**
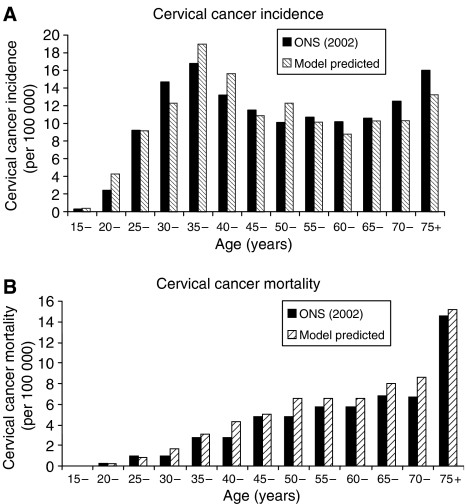
Comparison of model-predicted and observed data for age-specific cervical cancer incidence (**A**) and cervical cancer mortality (**B**).

Table 2 provides the transition probabilities that resulted from calibration. Results showed that model outputs compared well with UK data. The percentage deviations, calculated using age-specific rates as weights, were 9.7, 13.8 and 17.8% for HPV prevalence, cancer incidence and mortality, respectively. Results were similar when using other weighting methods (data not shown). The percentage deviation for overall CIN prevalence was 17.8%. Specifically, age-specific HPV prevalence was matched well to data from the Manchester study (Peto *et al*, 2004b), with a peak prevalence age range of 15–19 years (Figure 2A). Model-predicted CIN prevalence (CIN 1=0.8%, CIN 2=0.4%, CIN 3=0.7%) was comparable to data from the UK Screening Program (Department of Health Bulletin, 2004) (CIN 1=0.6%, CIN 2=0.5%, CIN 3=0.7%), with age-specific patterns similar to the Manchester study. The model closely reproduced HPV type distribution within normal cytology, cervical lesions and cancer, where values generally remained within the range observed from studies across the UK (Figure 2B, C, and D). The model predicted a crude average cervical cancer incidence rate of 10.5 per 100 000, compared with the reported rate of 10.6 per 100 000. Cervical cancer mortality also showed good correspondence (Figure 3) (ONS, 2002a, 2002b). Without screening, the model predicted an age-specific pattern of cervical cancer similar to historical rates (results not shown) (Parkin, 1966).

### Public health impact of vaccination

**Figure 4:**
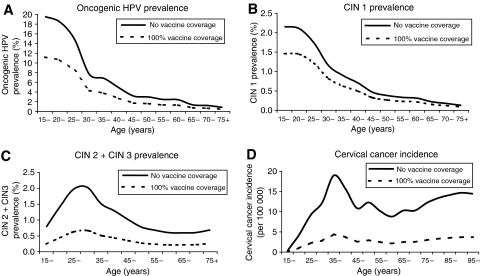
Impact of HPV 16/18 vaccine on HPV and cervical cancer epidemiology in the UK. (**A**) Oncogenic HPV prevalence; (**B**) CIN 1 prevalence; (**C**) CIN 2+CIN 3 prevalence; (**D**) cervical cancer incidence.

Over the lifetime of the cohort of 12-year-olds, the model predicts the occurrence of approximately 2 636 cervical cancer cases and 1 403 cancer deaths without vaccination, assuming that screening practices remain constant. With vaccination, the forecast would be as low as 632 cancer cases (76% reduction) and 335 cancer deaths (76% reduction). Figure 4D describes the projected impact of vaccination on the observed distribution of cervical cancer incidence across ages.

Vaccination was also predicted to give a considerably high reduction in the prevalence of high-grade lesions (i.e., CIN 2+CIN 3) across all ages (Figure 4C). In particular, vaccination was estimated to lead to a 95% reduction in the prevalence of lesions associated with HPV 16 and 18, consistent with what is assumed to be the type-specific vaccine efficacy rate. When considering lesions caused by all HPV types, the absolute prevalence of high-grade lesions is predicted to reduce from 1.07 to 0.36% (66% reduction) with vaccination. This estimated reduction of 66% falls within the model-predicted percentage of high-grade lesions attributable to HPV 16 and 18 and other oncogenic types (Figure 2).

It was also predicted that vaccination would have benefits in relation to HPV prevalence and low-grade lesions (CIN 1), although to a lesser extent. When considering only oncogenic HPV, the estimated percentage reduction in HPV prevalence was 45% with vaccination (Figure 4A). For all HPV types within the general population (oncogenic+low-risk), the projected reduction in HPV prevalence is lower at 35% (age-specific data not shown). The projected reduction in CIN 1 lesions with vaccination was 31% when considering CIN lesions caused by all HPV types, which is comparable to the projected reduction of overall HPV prevalence in the general population (Figure 4B).

As a consequence of the projected reductions in cervical disease owing to vaccination, clinical benefit is anticipated from a reduction in screening tests and treatments. Predicted numbers of abnormal cytology tests, diagnostic tests and treatments for precancerous lesions, occurring over the lifetime of a cohort of 12-year-old girls, with and without vaccination are shown in Table 3. With vaccination of the entire birth cohort, a reduction of close to 75 000 abnormal cytology tests is predicted. If the reduction in follow-up cytology tests that are normal is included, a total reduction of approximately 442 000 cytology tests is predicted with vaccination. The largest percentage reduction in screening and treatment events is observed in treatment of CIN lesions, approaching close to 43% reduction with 100% vaccination coverage.

### Sensitivity analyses

We investigated the impact of varying assumptions concerning vaccine efficacy and waning, vaccination coverage and age at vaccination, assuming 100% vaccination coverage. Table 4 details the results of these analyses for the more severe cervical outcomes. Despite the range of sensitivity analyses conducted, model predictions consistently suggested high efficacy of vaccination. The absolute prevalence of high-grade lesions ranged from 0.33% (69% reduction) to 0.53% (50% reduction) with different vaccination scenarios. Predictions for number of cases ranged from 538 cancer cases (80% reduction) to 1 032 cancer cases (61% reduction) and cancer deaths from 287 deaths (80% reduction) to 549 deaths (61% reduction) for different vaccination scenarios. Of all variables considered, results were most sensitive to age at vaccination and the vaccination coverage rate.

## DISCUSSION

The imminent global arrival of HPV vaccines with proven efficacy against HPV types 16 and 18 (Koutsky *et al*, 2002; Harper *et al*, 2004, 2006; Villa *et al*, 2005) means that decisions on adoption will soon need to be made across countries. These decisions are already being made within the United States, with the recent approval of Merck's HPV vaccine (Gardasil^®^). To facilitate such decisions, a detailed model of the natural history of HPV infection and cervical cancer was developed, capable of evaluating the long-term impact of a type-specific HPV vaccine in different countries with varying cervical cancer epidemiology and screening policies. The present study gives an example of this adaptation to the specific situation in the UK.

Calibration achieved a consistently close fit to HPV and CIN prevalence, as well as ICC incidence and mortality for the UK. Further, the model reflects the prevalence of HPV types within different stages of cervical disease, including low- and high-grade lesions, and ICC. Calibrating simultaneously across the spectrum of cervical disease, which is not a new concept (see e.g. Goldie *et al*, 2004), provides a robust foundation for the evaluation of the long-term impact of vaccination. Furthermore, we calibrated to data observed in the presence of screening, thus allowing use of the most recent epidemiological data in the UK. For some previous models, calibration was conducted to match data in unscreened women, which in the UK would have meant the use of older data before screening was introduced. Both in previous models and ours, calibration has been conducted using cross-sectional data (Canfell *et al*, 2004; Goldie *et al*, 2004; Sherlaw-Johnson and Philips, 2004; Taira *et al*, 2004). Ideally, we would have used longitudinal data as we are modelling a single cohort over time, but available UK data covered periods that were either too short or extended before and after the introduction of screening, making this impossible.

Perhaps the most striking model prediction is that 100% vaccination coverage of a 12-year-old cohort of girls would lead to a reduction of 66% in the prevalence of high-grade cervical lesions and a 76% reduction in cervical cancer deaths. These beneficial effects of vaccination are entirely plausible as they reflect the prevalence of HPV 16/18 in high-grade lesions and cervical cancer and the potential additional impact of prevention of HPV 45 and 31 infection (Clifford *et al*, 2003a, 2003b). A comparable study of vaccination against HPV 16 and 18 in the United States showed a reduction in ICC of 65 with 100% vaccine coverage (Goldie *et al*, 2004). The higher predicted reduction in our study is likely due to inclusion of efficacy against infection with HPV types 31 and 45, as well as a higher proportion of ICC associated with HPV 16 and 18 reported in UK studies. As HPV type distribution within cervical cancer varies geographically (Clifford *et al*, 2003b), it is expected that the benefits of HPV vaccination will vary by region.

Results also predict that approximately one-quarter of abnormal cytology tests and one-third of diagnostic tests and CIN treatments would be avoided with vaccination. This has important implications for reducing both NHS costs and the distress associated with positive tests (Bell *et al.*, 1999). Furthermore, we have assumed that introduction of a vaccine does not change current screening, whereas in practice it may be possible to reduce the burden of routine screening (Franco *et al*, 2006). Cost-effectiveness analyses are needed to explore optimal screening practice in combination with vaccination in the UK including the use of HPV testing in screening, triage and follow-up.

Results were sensitive to an increase in vaccination age, with projected benefits at 18 years reduced compared with 12 years. This is due to the pre-existing level of persistent HPV infection and related disease in the older adolescent and mature population and vaccination is predicted to provide maximum benefit at age 12, before sexual activity is initiated in most girls. Further, the projected benefit of vaccination was lower when assuming an 80% vaccination coverage rate, a rate similar to that observed in adolescent hepatitis B vaccination (Wallace *et al*, 2004). In this scenario, however, we have not considered the beneficial effects of herd immunity and would have thereby underestimated the full potential benefit. Assuming no cross-protection against types 31 and 45 also reduced the projected benefit of vaccination. This is important, as protection has only been demonstrated so far against incident infection for these types. Furthermore, assuming that protection against HPV types 31 and 45, though present, wanes to zero within 10 years, results in little extra benefit compared with assuming no cross protection, even if a booster is given after 10 years. However, this is an aggressively conservative waning profile and data from larger phase III studies is needed to establish the translation of protection against incident infection into prevention of persistent infection and precancerous lesions and the durability of protection for non-16/18 HPV types. Protection against all oncogenic types is most important up to age 25 years, when the risk of exposure to HPV is greatest, and it is possible that this exposure may naturally boost and maintain protection. However, boosters remain a possibility and their cost should be considered in cost-effectiveness analyses of HPV-16/18 vaccination.

There are limitations to our estimates of the beneficial effects of vaccination that should be considered. First, evidence suggests that HPV prevalence rates have increased in the UK since 1990 (Kitchener *et al*, 2004), which could mean that the burden of disease avoidable by vaccination is also increasing. We have neither included these higher rates in the analysis nor have we included the potential impact of vaccination on HPV-related neoplasms in other sites, such as the vulva, vagina, anus, penis and oropharynx. Second, through the effects of herd immunity, one would expect a marked decrease in HPV prevalence rates among males if girls were vaccinated. Thus, a secondary beneficial effect of vaccination, which is not taken into account in our analysis, would be to slow and perhaps even halt the growth of the HPV epidemic. Estimating the magnitude of the latter effect is beyond the scope of the present study. Third, the natural history of multiple HPV infections was not explicitly modelled. Therefore, the possibility that suppressing types 16 and 18 may allow the prevalence of other oncogenic HPV types in CIN and ICC to increase was not explored. Fourth, we have attempted to model the disease in enough detail to generate estimates of sufficient robustness to inform future health policy decisions. However, owing to the limits imposed by model complexity and the available data, as with previous modelling studies, it was not possible to explicitly account for all of the known heterogeneity in the cervical cancer disease process. Finally, as the model is calibrated to UK data, caution should be exercised in applying these findings to other regions with different screening practices and epidemiology.

In summary, mathematical models provide a means for extrapolating results beyond clinical trials and exploring the long-term impact of vaccination on outcomes. This analysis suggests that the public health benefits of HPV 16/18 vaccination, within the context of an effective screening programme, may be substantial in the UK, with large reductions not only in cervical cancer incidence and mortality, but also in the prevalence of precancerous lesions and associated diagnostic tests and treatments.

## Figures and Tables

**Table 1:** United Kingdom screening parameter model inputs

**Screening patterns**	**Value**	**Reference**
Start and stop age (years)	20–65	Department of Health Bulletin (2004)
		
*Screening coverage*		
% Screened every 3 years (dependent on age)	33–73	Department of Health Bulletin (2004)
% Never screened in lifetime	7	
		
*Test characteristics*		
Cytology – sensitivity (specificity)	0.41–0.67 (0.966)	
Probability of accurate biopsy CIN diagnosis	0.536	Cuzick *et al* (1995); Hopman *et al* (1998); Mitchell *et al* (1998)
Probability of biopsy underdiagnosed CIN lesion	0.2	
Probability of biopsy overdiagnosed CIN lesion	0.263	
Colposcopy – sensitivity (specificity)	0.96 (0.48)	
		
*Screening practices*		
Borderline dyskaryosis to triage cytology, (colposcopy) (%)	80 (20)	
Mild dyskaryosis to triage cytology, (colposcopy) (%)	58 (42)	Department of Health Bulletin (2004)
⩾Moderate dyskaryosis to colposcopy (%)	100	
Negative triage cytology to regular screening (repeat test) (%)	84 (16)	Assumption/(NHS, 2004)
Positive triage cytology to colposcopy (%)	100	
Negative colposcopy/biopsy to regular screening (%)	50	
Negative colposcopy/biopsy to increased screening (%)	50	
CIN 1 diagnosis to increased screening, (treatment) (%)	50 (50)	
CIN 2 or 3 diagnoses to treatment (%)	100	

CIN = cervical intraepithelial neoplasia; Cytology sensitivity = probability of abnormal cytology given true state is CIN 1+. The model includes probability of abnormal cytology according to lesion type (i.e., CIN 1 to CIN 3) and therefore a range of values is provided; cytology specificity = probability of normal cytology given true state is negative for lesions.

**Table 2:** Six-month transition probabilities used in the United Kingdom model calibration

**Variable**		**Oncogenic HPV**	**Nononcogenic HPV**	**References**
*Lesion progression and regression*				
Normal to HPV	<35 years	0.023–0.077	0.008–0.026	*HPV acquisition and regression*; Franco *et al* (1999b); Moscicki *et al* (2001); Molano *et al* (2003); Schlecht *et al* (2003)
	>35 years	0.004–0.023	0.001–0.008	
HPV to CIN 1	<35 years	0.045–0.050	0.037–0.042	*Lesion progression and regression:* Ho *et al* (1998); Melnikov *et al* (1998); Holowaty *et al* (1999); Schlecht *et al* (2003); Cantor *et al* (2004)
	>35 years	0.05	0.042	
CIN 1 to CIN 2	<35 years	0.014–0.278	0.007–0.017	
	>35 years	0.035–0.315	0.017–0.020	
CIN 2 to CIN 3	<35 years	0.100–0.185	0.100–0.185	
	>35 years	0.185–0.200	0.185–0.200	
HPV clearance	<35 years	0.38	0.53	
	>35 years	0.38	0.53	
CIN 1 regression	<35 years	0.340–0.440	0.380–0.480	
	>35 years	0.31	0.32	
CIN 2/3 regression	<35 years	0.02	0.02	
	>35 years	0.02	0.02	
				
*Progression to invasive cancer*				
CIN 3 to Cancer		0.002–0.017	0.008	*Natural history of invasive cancer*: De Rijke *et al* (2002); Goldie *et al* (2004)
				
*Stage I*				
Progression to stage II		0.11	0.11	
Probability of symptoms		0.075	0.075	
Mortality		0.005–0.015	0.005–0.015	
				
*Stage II*				
Progression to stage III		0.12	0.12	
Probability of symptoms		0.113	0.113	
Mortality		0.015–0.040	0.015–0.040	
				
*Stage III*				
Progression to stage IV		0.12	0.12	
Probability of symptoms		0.3	0.3	
Mortality		0.050–0.090	0.050–0.090	
				
*Stage IV*				
Probability of symptoms		0.45	0.45	
Mortality		0.070–0.120	0.070–0.120	

Ranges are reported owing to probability variation in age and HPV type. References are provided that support the resulting transition probability values.

CIN (cervical intraepithelial neoplasia) 1 lesions can regress to HPV (human papillomavirus) infection or normal; CIN2/3 lesions can regress to HPV infection or normal. Details of the point estimates from the calibrated model are available from the authors upon request.

**Table 3:** Impact of HPV 16/18 vaccine on abnormal cytology, diagnostic tests, and treated CIN lesions over the lifetime of a 12-year-old cohort in the United Kingdom

	**Abnormal cytology test^a^**	**Colposcopy**	**Biopsy**	**Treated CIN lesions**
0% vaccine coverage				
	311 983	153 245	99 974	38 437
				
100% vaccine coverage				
	237 734	111 504	67 367	22 123
				
Reduction owing to vaccine	74 249	41 741	32 607	16 314
% Reduction	23.8%	27.2%	32.6%	42.4%

a Abnormal cytology test includes those with borderline dyskaryosis or greater.

**Table 4:** The impact of alternative assumptions for vaccine efficacy, waning, and vaccination age on selected cervical cancer outcomes in the United Kingdom

	**CIN 2+CIN 3 prevalence**	**Cervical cancer cases (*n*)**	**Cervical cancer deaths (*n*)**
No vaccine	1.07%	2636	1403
			
*Base case* a			
	0.361%	632	335
% Reduction	66.3%	76.0%	76.1%
			
*Scenario 1: Low efficacy (HPV 16/18)*			
	0.390%	724	384
% Reduction	63.6%	72.5%	72.7%
			
*Scenario 2: High efficacy (HPV 16/18)*			
	0.331%	538	287
% Reduction	69.0%	79.6%	79.6%
			
*Scenario 3: No cross protection*			
	0.410%	710	375
% Reduction	61.7%	73.1%	73.3%
			
*Scenario 4: Vaccination coverage (80%)*			
	0.502%	1032	549
% Reduction	53.1%	60.8%	60.9%
			
*Scenario 5: Lower age at vaccination (10 years)*			
	0.361%	631	335
% Reduction	66.3%	76.0%	76.1%
			
*Scenario 6: Higher age at vaccination (18 years)*			
	0.535%	896	506
% Reduction	50.0%	66.0%	63.9%
			
*Scenario 7: Vaccine waning (HPV 31, 45 types)*			
	0.409%	709	375
% Reduction	61.8%	73.1%	73.3%
			
*Scenario 8: Vaccine waning (HPV 31, 45 types)+Booster at 10 years*			
	0.400%	698	368
% Reduction	62.7%	73.5%	73.8%
			
*Scenario 9: Decreased HPV type distribution of 16/18 in cervical cancer*			
	0.356%	749	397
% Reduction	66.7%	71.6%	71.7%

a Base case assumes 95% efficacy against 16 and 18 infection, 50% efficacy against HPV 31, 90% efficacy against HPV 45. No waning is assumed. Results are provided for 100% vaccine coverage.
